# Exacerbation history and blood eosinophil count prior to diagnosis of COPD and risk of subsequent exacerbations

**DOI:** 10.1183/13993003.02240-2023

**Published:** 2024-10-03

**Authors:** David M.G. Halpin, Heath Healey, Derek Skinner, Victoria Carter, Rachel Pullen, David Price

**Affiliations:** 1 University of Exeter Medical School, College of Medicine and Health, University of Exeter, Exeter, UK; 2 Observational and Pragmatic Research Institute, Singapore, Singapore; 3 Optimum Patient Care, Cambridge, UK; 4 Centre of Academic Primary Care, Division of Applied Health Sciences, University of Aberdeen, Aberdeen, UK

## Abstract

**Background:**

Prior exacerbation history is used to guide initial maintenance therapy in COPD; however, the recommendations were derived from patients already diagnosed and treated.

**Methods:**

We assessed the rates of moderate (*i.e.* treated with antibiotics and/or systemic corticosteroids) and severe (*i.e.* hospitalised) exacerbations in the year following diagnosis in patients newly diagnosed with COPD according to their prior history of exacerbations, blood eosinophil count (BEC) and whether maintenance therapy was started. Data were extracted from the Optimum Patient Care Research Database.

**Results:**

73 189 patients were included. 61.9% had no exacerbations prior to diagnosis, 21.5% had 1 moderate, 16.5% had ≥2 moderate and 0.3% had ≥1 severe. 50% were started on maintenance therapy. In patients not started on maintenance therapy the rates of moderate exacerbations in the year after diagnosis in patients with no, 1 moderate, ≥2 moderate and ≥1 severe prior exacerbations were 0.34 (95% CI 0.33–0.35), 0.59 (95% CI 0.56–0.61), 1.18 (95% CI 1.14–1.23) and 1.21 (95% CI 0.73–1.69), respectively. Similar results were seen in patients started on maintenance therapy. BEC did not add significantly to the prediction of future exacerbation risk.

**Conclusions:**

A single moderate exacerbation in the year prior to diagnosis increases the risk of subsequent exacerbations, and more frequent or severe exacerbations prior to diagnosis are associated with a higher risk.

## Shareable abstract

**In newly diagnosed patients, a single exacerbation prior to diagnosis of COPD is associated with a significant risk of exacerbations over the next 12 months, and more frequent or severe exacerbations prior to diagnosis are associated with a higher risk** https://bit.ly/4fhTSOm

## Graphical abstract

**Figure:**
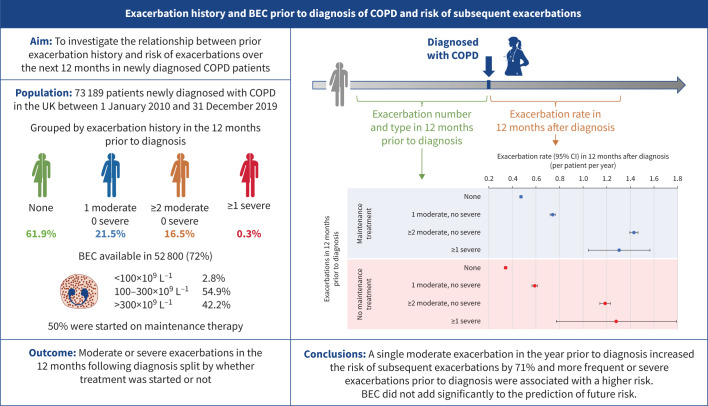
Overview of the study. BEC: blood eosinophil count.

## Introduction

Reducing the risk of exacerbations is a key objective of COPD management [1] given the substantial evidence about the relationship between exacerbations and poor clinical outcomes, including death [2–5]. The Global Initiative for Chronic Obstructive Lung Disease (GOLD) recommends assessing patients’ risk of exacerbations at the initial assessment by considering the number and severity of exacerbations in the previous year. The GOLD Report advises combining this risk assessment with the blood eosinophil count (BEC) to determine the most appropriate initial pharmacological therapy [1].

GOLD recommends that patients should be considered at “high risk” of exacerbations if they had ≥2 moderate exacerbations (*i.e.* treated with antibiotics and/or systemic corticosteroids but not hospitalised) or 1 severe (*i.e.* hospitalised) exacerbation in the previous 12 months. The threshold was chosen by reference to data from the ECLIPSE (Evaluation of COPD Longitudinally to Identify Predictive Surrogate Endpoints) study [6]; however, the patients in that study were not newly diagnosed, 76% were on long-acting bronchodilators and 72% were on inhaled corticosteroids (ICS). Although there is considerable evidence about the relationship between the history of exacerbations and future risk in patients with a diagnosis of COPD and on treatment [6–10], there is a paucity of evidence in untreated and newly diagnosed patients.

This study aimed to determine the relationship between the frequency and severity of exacerbations in the year prior to diagnosis and the rate of exacerbations in the year after diagnosis in patients newly diagnosed with COPD in a community setting between 2010 and 2019, stratified by whether they were started on maintenance therapy and by BEC. We tested the hypothesis that a single moderate exacerbation in the year prior to diagnosis is associated with an increased rate of exacerbations after diagnosis.

## Methods

### Study design and data sources

Data were sourced from the Optimum Patient Care Research Database (OPCRD; https://opcrd.co.uk), which contains anonymised routine, patient-level diagnostic, clinical and prescribing information on over 24 million patients from over 1000 primary care centres across the UK (∼35% of the total UK population) [11]. OPCRD has received a favourable opinion from the NHS Health Research Authority for anonymous research use (REC reference: 15/EM/0150). Governance is provided and the study protocol was approved by the Anonymised Data Ethics and Protocols Transparency committee (ADEPT0923), an independent body of experts and regulators commissioned by the Respiratory Effectiveness Group (www.effectivenessevaluation.org). The study protocol was established prior to data extraction, in accordance with the criteria for the European Network Centres for Pharmacoepidemiology and Pharmacovigilance (ENCePP; www.encepp.eu), and is registered with the ENCePP (EUPAS107087).

Patients had to have a first diagnosis of COPD (at least one diagnostic COPD Read code (Quality and Outcomes Framework defined)) between 1 January 2010 and 31 December 2019, at least 12 months of continuous primary care data prior to the date of diagnosis and at least 12 months of follow-up post-diagnosis. Patients had to have a forced expiratory volume in 1 s/forced vital capacity ratio <0.7 and no other chronic respiratory conditions. A prior diagnosis of asthma was not used as an exclusion criterion as it is often confused with COPD before a definitive diagnosis is made and can also be a comorbidity of COPD. The study variables and outcomes collected are listed in the supplementary material.

Exacerbations were identified as in previous studies [12, 13], and their severity was defined as moderate if they were associated with prescription of an antibiotic or oral steroids and severe if there was admission to hospital. Patients were grouped into four cohorts based on the exacerbation history in the 12 months prior to diagnosis: none, 1 moderate and no severe, ≥2 moderate and no severe, and ≥1 severe. They were also stratified according to whether maintenance treatment was started at diagnosis and by BEC (<100, 100–300 and >300×10^9^ L^−1^).

### Statistical analysis

Kruskal–Wallis tests followed by Dunn's test were used to compare distributions of modified Medical Research Council (mMRC) dyspnoea scores, COPD Assessment Test (CAT) scores and BEC, and Chi-squared to compare the rates of initiation of maintenance therapy between prior exacerbation groups. Negative binomial regression was used to investigate the association between prior exacerbation frequency and severity and the rate of subsequent exacerbations. Incidence rate ratios (IRRs) compared to patients with no prior exacerbations and their 95% confidence intervals were calculated. Non-parametric trend test nptrend was used to compare rates of exacerbations across patients grouped by exacerbation history and BEC. Analyses were conducted using Stata/SE version 14.2 (StataCorp, College Station, TX, USA). A two-sided significance level α of 0.05 was defined and Bonferroni adjustment was carried out to allow for the multiple comparisons.

## Results

**FIGURE 1:**
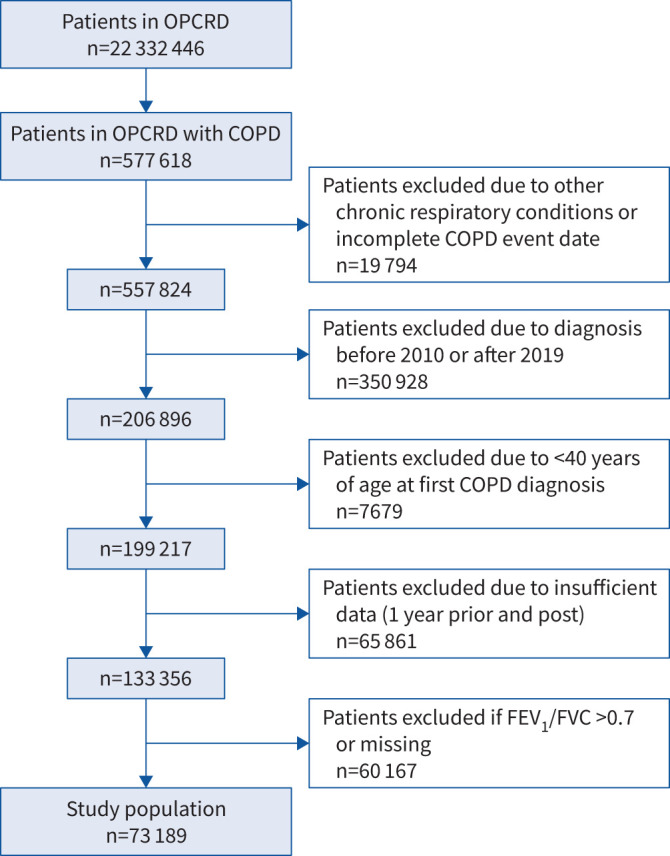
Patient flow. OPCRD: Optimum Patient Care Research Database; FEV_1_: forced expiratory volume in 1 s; FVC: forced vital capacity.

73 189 patients met the eligibility criteria and were included (figure 1). In the 12 months prior to diagnosis, 45 205 (61.9%) had not had an exacerbation, 15 717 (21.5%) had 1 moderate but no severe exacerbations, 12 063 (16.5%) had ≥2 moderate but no severe exacerbations and 204 (0.3%) had ≥1 severe exacerbations. 2857 (3.9%) of patients had 3 moderate exacerbations in the year prior to diagnosis and 2868 (3.9%) had ≥4. 192 (0.3%) patients had 1 severe exacerbation in the year prior to diagnosis, 10 had 2 severe exacerbations and two had 3 severe exacerbations. None had >3 severe exacerbations in the year prior to diagnosis.

The baseline characteristics of the patients are presented in table 1. Overall, 36 831 (50%) patients were not started on maintenance therapy at the time of diagnosis. BEC was available in 52 800 (72%) patients.

**TABLE 1:** Baseline characteristics of patients according to prior exacerbation history

	Exacerbations in 12 months prior to diagnosis			
	None	1 moderate, no severe	≥2 moderate, no severe	≥1 severe
**Patients**	45 205	15 717	12 063	204
**Age (years)**	66.38±10.56	66.09±10.61	66.23±10.99	64.71±11.58
**Sex**				
Male	25 530 (57)	8230 (57)	6320 (53)	99 (53)
Female	19 456 (43)	7380 (43)	5675 (47)	104 (47)
Missing	219 (0.5)	107 (0.7)	68 (0.6)	1 (0.5)
**BMI (kg·m^−2^)^#^**	27.08±5.85	27.25±6.02	27.80±6.11	28.31±7.25
Missing	2273 (5.0)	788 (5.0)	524 (4.3)	9 (4.4)
**Smoking status** ^¶^				
Current smoker	11 217 (27.0)	3781 (26.4)	2388 (21.6)	39 (21.5)
Ex-smoker	25 871 (62.4)	9077 (63.5)	7193 (65.2)	113 (62.4)
Non-smoker	4403 (10.6)	1440 (10.1)	1457 (13.2)	29 (16.0)
Missing	3714 (8.2)	1419 (9.0)	1025 (8.4)	23 (11.3)
**GOLD stage^+^**				
1	596 (2.2)	242 (2.5)	182 (2.5)	3 (2.8)
2	4416 (16.5)	1720 (17.8)	1332 (18.2)	20 (18.9)
3	15 208 (57.0)	5681 (58.7)	4301 (58.6)	70 (66.0)
4	6484 (24.3)	2036 (21.0)	1520 (20.7)	13 (12.3)
Missing	18 501 (40.9)	6038 (38.4)	4728 (39.2)	98 (48.0)
**GOLD group^§^**				
A	29 819 (71.2)	10 363 (70.8)	0 (0)	0 (0)
B	12 047 (28.8)	4273 (29.2)	0 (0)	0 (0)
E	0 (0)	0 (0)	12 063 (100)	204 (100)
Missing	3339 (7.4)	1081 (6.9)	()	()
**mMRC dyspnoea score**				
0	10 503 (25.1)	3607 (24.7)	2322 (20.6)	26 (13.7)
1	18 777 (44.9)	6597 (45.1)	5053 (44.9)	74 (38.9)
2	8960 (21.4)	3180 (21.7)	2708 (24.1)	52 (27.4)
3	3144 (7.5)	1094 (7.5)	997 (8.9)	30 (15.8)
4	449 (1.1)	148 (1.0)	166 (1.5)	8 (4.2)
Missing	3372 (7.5)	1091 (6.9)	817 (6.8)	14 (6.9)
**CAT score^ƒ^**				
Normal: 0– <6	1509 (18.7)	455 (16.9)	272 (13.0)	5 (9.1)
Low: 6– <10	1736 (21.6)	592 (22.0)	372 (17.8)	10 (18.2)
Medium: 10– <21	3481 (43.2)	1152 (42.7)	925 (44.4)	27 (49.1)
High: 21– <31	1101 (13.7)	412 (15.3)	412 (19.8)	8 (14.5)
Very high: 31– <41	227 (2.8)	84 (3.1)	104 (5.0)	5 (9.1)
Missing	37 151 (82.2)	13 022 (82.9)	9978 (82.7)	149 (73.0)
**BEC (×10^9^ L^−1^)^##^**	210 (160–350)	230 (180–390)	260 (190–400)	220 (190–400)
**Grouped BEC^##^**				
<100×10^9^ L^−1^	965 (3.0)	341 (3.1)	219 (2.5)	6 (4.0)
100–300×10^9^ L^−1^	18 381 (56.4)	6003 (53.8)	4534 (51.1)	85 (56.7)
>300×10^9^ L^−1^	13 268 (40.7)	4818 (43.2)	4121 (46.4)	59 (39.3)
Missing	12 591 (27.9)	4555 (29.0)	3189 (26.4)	54 (26.5)
**Initial treatment** ^¶¶^				
LABA	2012 (4.5)	774 (4.9)	636 (5.3)	14 (6.9)
LAMA	10 309 (22.8)	4160 (26.5)	3572 (29.6)	67 (32.8)
LABA/ICS	8237 (18.2)	3363 (21.4)	3625 (30.1)	80 (39.2)
LABA/LAMA	1703 (3.8)	557 (3.5)	322 (2.7)	6 (2.9)
LABA/LAMA/ICS	118 (0.3)	51 (0.3)	50 (0.4)	1 (0.5)
None of the above^++^	24 487 (54.2)	7518 (47.8)	4764 (39.5)	62 (30.4)
**Prevalence of comorbidities prior to initial COPD diagnosis**				
Asthma	9988 (22.1)	4286 (27.3)	4950 (41.0)	150 (73.5)
Hypertension	4812 (10.6)	2032 (12.9)	2451 (20.3)	74 (36.3)
Ischaemic heart disease	987 (2.2)	462 (2.9)	559 (4.6)	12 (5.9)
Heart failure	966 (2.1)	456 (2.9)	549 (4.6)	17 (8.3)
Chronic kidney disease	1735 (3.8)	720 (4.6)	873 (7.2)	16 (7.8)
Type 2 diabetes	1779 (3.9)	762 (4.8)	1042 (8.6)	28 (13.7)
Osteoporosis	1049 (2.3)	494 (3.1)	738 (6.1)	24 (11.8)
Depression or anxiety	4678 (10.3)	2253 (14.3)	2775 (23.0)	82 (40.2)

Data are presented as n, mean±sd, n (%) or median (interquartile range). All percentages relate to patients with known values; those with missing values were excluded from the calculations. BMI: body mass index; GOLD: Global Initiative for Chronic Obstructive Lung Disease; mMRC: modified Medical Research Council; CAT: COPD Assessment Test; BEC: blood eosinophil count; LABA: long-acting β-agonist; LAMA: long-acting muscarinic antagonist; ICS: inhaled corticosteroid. ^#^: BMI recorded or calculated BMI nearest (within 2 years) to initial COPD diagnosis; ^¶^: smoking status nearest (before or after) initial COPD diagnosis; ^+^: GOLD stage based on forced expiratory volume in 1 s percentage predicted (within up to 5 years prior); ^§^: GOLD 2023 grouping A, B and E; ^ƒ^: CAT score nearest (within 1 year) to initial COPD diagnosis; ^##^: maximum eosinophil reading within 1 year of initial COPD diagnosis; ^¶¶^: patients may have received multiple drugs on the same “initial treatment” day, combination product counts do not include multiple single items on the same day; ^++^: patients classed as none may receive other drug classes not included in this list, *e.g.* short-acting β-agonists, *etc.*

### Baseline characteristics according to prior exacerbation history

There was no significant difference in body mass index according to prior exacerbation history (table 1). There was a higher proportion of current smokers in patients who had not had an exacerbation (27.0%) or only 1 moderate exacerbation (26.4%) compared to those with more frequent (21.6%) or severe exacerbations (21.5%) (table 1). mMRC dyspnoea scores were higher in patients with more frequent or severe exacerbations prior to diagnosis (p=0.001) (table 1). Although there were many patients who did not have a CAT score recorded, among those who did, a lower proportion of patients who had previously had a severe exacerbation had normal or low CAT scores [14] and a higher proportion with frequent or severe exacerbations had medium, high or very high CAT scores (p=0.001) (table 1). Compared to those with ≥1 exacerbations, fewer patients with no previous exacerbations were started on maintenance therapy at diagnosis, and the proportion treated rose with increasing exacerbation frequency and severity (p<0.001) (table 1). Within each prior exacerbation group, more of the patients started on maintenance therapy had high CAT and mMRC dyspnoea scores compared to those not started on therapy (supplementary table S1). Otherwise, there were no differences in the baseline characteristics of patients started or not started on maintenance therapy (supplementary table S1).

When medication was started, there was no pattern in the different classes of maintenance therapy used and prior exacerbation history (table 1). Long-acting muscarinic antagonist (LAMA) monotherapy and long-acting β-agonist (LABA)/ICS therapy were the most commonly started therapies. Triple therapy with LABA/LAMA/ICS was the initial maintenance therapy in <0.5% of patients. The types of maintenance treatment started over time are shown in supplementary figure S1. Apart from the low-level use of LABA/LAMA and LABA/LAMA/ICS combinations following their licensing, there were no clear differences in use over time in any of the groups.

The proportions of patients with comorbidities diagnosed prior to the diagnosis of COPD were higher in patients with more frequent moderate or severe exacerbations (table 1). The proportions of patients with comorbidities were higher in those started on maintenance therapy within each prior exacerbation category (supplementary table S1).

BECs were not normally distributed in any of the prior exacerbation groups (supplementary figure S2). Overall, 1531 of patients had BEC <100×10^9^ L^−1^ (2.8% of those with values available), 29 003 (54.9%) had BEC 100–300×10^9^ L^−1^ and 22 266 (42.2%) had BEC >300×10^9^ L^−1^. The median BEC value was significantly higher in patients with ≥2 moderate exacerbations prior to diagnosis than in those with 1 moderate exacerbation (table 1), and the median BEC in both of these groups was significantly higher than in patients who had not had an exacerbation (p<0.001 for all). There were no significant differences between BEC in patients with ≥1 severe exacerbations and the other groups, probably reflecting the smaller number of patients in this group. A higher proportion of patients had BEC >300×10^9^ L^−1^ in the more frequent and more severe prior exacerbation groups than in patients with only 1 moderate or no prior exacerbations (p=0.001) (table 1).

### Exacerbation rates in the year after diagnosis

12.9% of patients had ≥1 moderate exacerbations in the year after diagnosis, but only 0.26% of patients had severe exacerbations. When assessing the relationship between exacerbation history and risk of future exacerbation rates we combined both moderate and severe events when calculating the rates post-diagnosis.

**FIGURE 2:**
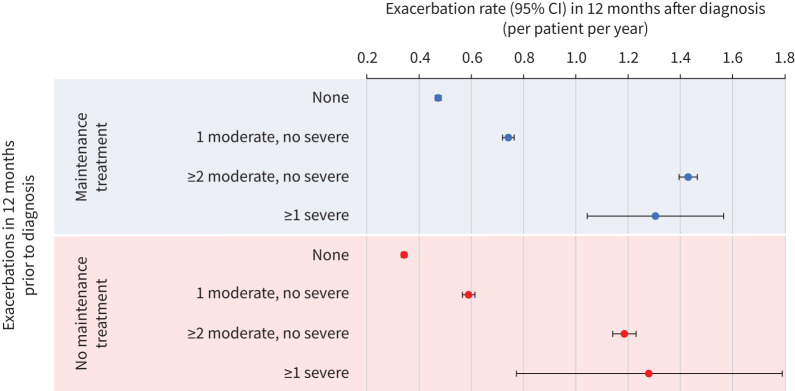
Rates of moderate or severe exacerbations in the 12 months after diagnosis with 95% confidence intervals, according to exacerbation history in the 12 months prior to diagnosis and whether maintenance therapy was started.

The rates of moderate or severe exacerbations, per patient per year, in the 12 months after diagnosis according to the exacerbation history in the year prior to diagnosis and whether maintenance therapy was started are shown in figure 2. The rates of exacerbations were significantly higher in patients with only 1 moderate exacerbation in the prior year compared to the rates in patients who had not had any in the prior year, irrespective of whether patients were prescribed maintenance therapy (p<0.01 for both). The rate was significantly higher again in those having ≥2 moderate exacerbations (p<0.01) and in those having ≥1 severe exacerbations in the prior year in both treated and untreated patients.

### Exacerbation rates in the year after diagnosis according to baseline BEC and whether maintenance therapy was started

The rates of moderate or severe exacerbations in the 12 months after diagnosis according to the exacerbation history in the year prior to diagnosis and BEC, together with the IRRs compared to patients with no prior exacerbations, are shown in table 2 (for patients not started on maintenance therapy) and table 3 (for patients started on maintenance therapy).

**TABLE 2:** Rate of moderate and severe exacerbations in the 12 months following diagnosis and incidence rate ratio (IRR) compared to patients with no prior exacerbations, in patients not started on maintenance therapy according to history of exacerbations in the 12 months prior to diagnosis and blood eosinophil count (BEC)

Exacerbation history in year prior to diagnosis	Patients (n)	Moderate or severe exacerbations in year following diagnosis	
**None**			
All	20 938	Rate	0.34 (0.33–0.35)
**1 moderate, no severe**			
All	6240	Rate	0.59 (0.57–0.61)
		IRR *versus* none	1.71 (1.66–1.76)
BEC <100×10^9^ L^−1^	161	Rate	0.50 (0.37–0.63)
		IRR *versus* none	1.46 (1.22–1.75)
BEC 100–300×10^9^ L^−1^	2388	Rate	0.56 (0.52–0.59)
		IRR *versus* none	1.62 (1.55–1.69)
BEC >300×10^9^ L^−1^	1795	Rate	0.67 (0.62–0.72)
		IRR *versus* none	1.95 (1.85–2.05)
**≥2 moderate, no severe**			
All	3878	Rate	1.19 (1.14–1.23)
		IRR *versus* none	3.44 (3.33–3.56)
BEC <100×10^9^ L^−1^	75	Rate	1.13 (0.79–1.48)
		IRR *versus* none	3.29 (2.63–4.2)
BEC 100–300×10^9^ L^−1^	1471	Rate	1.08 (1.01–1.15)
		IRR *versus* none	3.14 (2.98–3.32)
BEC >300×10^9^ L^−1^	1279	Rate	1.34 (1.26–1.43)
		IRR *versus* none	3.90 (3.69–4.13)
**≥1 severe**			
All	43	Rate	1.28 (0.77–1.79)
		IRR *versus* none	3.72 (2.76–5.01)
BEC <100×10^9^ L^−1^	1	Rate	NC
		IRR *versus* none	NC
BEC 100–300×10^9^ L^−1^	16	Rate	0.94 (0.06–1.81)
		IRR *versus* none	0.02 (0.01–0.03)
BEC >300×10^9^ L^−1^	15	Rate	1.13 (0.48–1.78)
		IRR *versus* none	2.92 (1.76–4.85)

95% confidence intervals are provided for rates and IRRs. NC: not calculable.

**TABLE 3:** Rate of moderate and severe exacerbations in the 12 months following diagnosis and incidence rate ratio (IRR) compared to patients with no prior exacerbations, in patients started on maintenance therapy according to history of exacerbations in the 12 months prior to diagnosis and blood eosinophil count (BEC)

Exacerbation history in year prior to diagnosis	Patients (n)	Moderate or severe exacerbations in year following diagnosis	
**None**			
All	24 267	Rate	0.47 (0.46–0.48)
**1 moderate, no severe**			
All	9477	Rate	0.74 (0.72–0.76)
		IRR *versus* none	1.57 (1.53–1.61)
BEC <100×10^9^ L^−1^	180	Rate	0.69 (0.53–0.85)
		IRR *versus* none	1.96 (1.65–2.32)
BEC 100–300×10^9^ L^−1^	3615	Rate	0.73 (0.69–0.76)
		IRR *versus* none	1.58 (1.52–1.64)
BEC >300×10^9^ L^−1^	3203	Rate	0.82 (0.78–0.86)
		IRR *versus* none	1.56 (1.49–1.62)
**≥2 moderate, no severe**			
All	8185	Rate	1.43 (1.39–1.46)
		IRR *versus* none	3.44 (3.35–3.53)
BEC <100×10^9^ L^−1^	144	Rate	1.51 (1.24–1.78)
		IRR *versus* none	4.3 (3.58–5.18)
BEC 100–300×10^9^ L^−1^	3063	Rate	1.37 (1.31–1.42)
		IRR *versus* none	2.96 (2.84–3.25)
BEC >300×10^9^ L^−1^	2842	Rate	1.58 (1.52–1.65)
		IRR *versus* none	3.01 (2.88–3.31)
**≥1 severe**			
All	161	Rate	1.3 (1.04–1.57)
		IRR *versus* none	2.76 (2.36–3.22)
BEC <100×10^9^ L^−1^	5	Rate	0.6 (0.12–1.08)
		IRR *versus* none	1.71 (0.71–4.12)
BEC 100–300×10^9^ L^−1^	69	Rate	1.16 (0.81–1.51)
		IRR *versus* none	2.52 (1.98–3.19)
BEC >300×10^9^ L^−1^	44	Rate	1.7 (1.1–2.31)
		IRR *versus* none	3.24 (2.41–4.36)

95% are provided confidence intervals for rates and IRRs.

In patients not started on maintenance therapy, there was no significant relationship between the rate of exacerbations in the year following diagnosis and BEC (table 2). In all BEC groups within each prior exacerbation group, the rates were higher than in patients who had not had an exacerbation. In patients who were started on maintenance therapy, there was also no significant relationship between BEC and the rates of exacerbations in the year following diagnosis, and in all BEC groups within each prior exacerbation group the rates were higher than in patients who had not had an exacerbation (table 3). In each prior exacerbation group, the rates of exacerbations in the 12 months after the diagnosis were similar in patients who were started on maintenance therapy containing ICS compared to those started on maintenance therapy not containing ICS. The rates were higher in patients with 1 moderate exacerbation compared to none, and higher again in those with ≥2 moderate or ≥1 severe exacerbations, irrespective of whether their treatment contained ICS (supplementary table S2).

## Discussion

This analysis of over 73 000 patients newly diagnosed with COPD in the UK between 2010 and 2019 provides important information to guide a personalised approach to optimal management based on individualised assessment of future exacerbation risk.

In patients who were not started on maintenance therapy, a single moderate exacerbation prior to diagnosis was associated with a 71% increase in risk of exacerbations in the year after diagnosis. In treated patients, a single moderate exacerbation prior to diagnosis was associated with a 57% increased risk of exacerbations. Using the threshold GOLD currently recommends [1], 16.8% of our patients would be in group E and classed at higher risk of future exacerbations. Our data support including patients with a single moderate exacerbation in group E and if the definition was changed to include these patients, 38.2% of our patients would fall into the higher risk group.

The relationship between exacerbation history prior to diagnosis and the risk of exacerbations over the next 12 months is likely to have been affected by maintenance therapy, as the treatments initiated have all been shown to reduce the rates of exacerbations [1]. We therefore explored the relationship between the exacerbation history in the year prior to diagnosis and the risk of exacerbations separately in treated and untreated patients and consider that the most relevant data about risk come from untreated patients. No conclusions can be drawn from comparisons of the rates between treated and untreated patients grouped by prior exacerbation history as the two groups are clearly different and therapy was started for a reason.

Therapy, in particular LABA/ICS, was more commonly started in patients with ≥2 moderate or ≥1 severe exacerbations prior to diagnosis, suggesting that clinicians took the exacerbation history into account when deciding whether to start therapy. Other factors may also have influenced the decision to start therapy and it is notable that within each prior exacerbation group the mMRC dyspnoea and CAT scores were higher in patients started on maintenance therapy, suggesting that symptoms may also have influenced the decision to start therapy.

In agreement with other studies, many of the patients had current or prior diagnoses of other conditions at the time they were diagnosed with COPD, most commonly asthma, hypertension and depression or anxiety. Comorbidities were more common in patients with more frequent or more severe prior exacerbations. Patients with a prior diagnosis of asthma may have had asthma when they were younger, they may have still had asthma at the time a diagnosis of COPD was made or they may have been misdiagnosed with asthma before it was realised that the correct diagnosis was COPD. All patients with asthma had spirometry confirming fixed airflow obstruction, and a clinical diagnosis of COPD had been made by their doctor and documented in their medical records. We think it is unlikely that any of the patients not started on maintenance therapy had current asthma.

There is conflicting evidence about the predictive value of BEC and exacerbation rates in diagnosed and treated patients, with studies reporting either no relationship [15] or a positive relationship [16, 17]; however, their interpretation is confounded by different prior exacerbation histories and use of ICS [18]. In our newly diagnosed patients, BEC was related to the prior exacerbation rate, but did not add significantly to the prediction of future exacerbation risk, even in those patients not started on maintenance therapy. The median BEC in our patients was similar to the mean values reported in other studies, but the distributions were skewed and more patients had BEC >300×10^9^ L^−1^ than has been reported in other studies. This may, at least in part, be due to the fact that we used the highest count within 12 months of the diagnosis rather than the mean of available values. As commonly found in primary care data, there was also evidence of rounding of BEC results to the nearest 100 (supplementary figure S2), which means some patients with BEC <100×10^9^ L^−1^ were included in the 100–300×10^9^ L^−1^ category. Irrespective of its ability to predict exacerbation risk, BEC is recommended to guide ICS therapy [18]. GOLD currently recommends considering including ICS in the initial therapy for patients in group E if the BEC is >300×10^9^ L^−1^ [1] on the pragmatic basis that ICS are more effective at reducing exacerbation rates in patients with higher BEC [19–23]. 46% of the patients in group E, as currently defined, had BEC >300×10^9^ L^−1^ and if patients with a single moderate exacerbation were also included in this group, 45% of all the patients in the expanded group E would have BEC >300×10^9^ L^−1^.

Whittaker *et al*. [24] have also looked at the association between the frequency and severity of lower respiratory tract infections (LRTIs) prior to diagnosis and the risk of future exacerbations using English primary care data, but our analysis differs from theirs in several important ways. They studied patients who were diagnosed between 2005 and 2019 and assessed exacerbation rates post-diagnosis for as long as follow-up data allowed. The median follow-up was 4.4 years and no account was taken of medication started at diagnosis or subsequently. Significant changes in clinicians’ awareness of COPD, recognition of the importance of preventing exacerbations and introduction of effective therapies occurred during the period 2004–2010 [13] and secular changes in exacerbation rates have been observed [25], which is why we restricted our analysis to patients diagnosed more recently. Whittaker *et al*. [24] did not assess the effect of BEC. Despite these differences, their findings support our finding that a single moderate exacerbation prior to diagnosis is associated with an increased risk of future events.

Our study has several strengths, including the large sample size, completeness of follow-up, the robustness of the COPD diagnosis in OPCRD which is based on symptoms and spirometry, the use of a validated algorithm to identify exacerbations, and the inclusion of BEC. OPCRD is a well-maintained database which is used frequently for observational research in COPD [13, 26–28], and it contains information which is collected prospectively and not subjected to recall bias. The dataset draws from a geographically and socioeconomically diverse population, and therefore we believe that the study results show the true relationship between exacerbation history and subsequent risk of moderate and severe exacerbations in newly diagnosed patients in the UK.

Potential study limitations include the fact that the dataset reflects the demographics of patients in the UK only. Individual patient records contain information collected for routine clinical use rather than for research purposes and as a result there are some missing data. Exacerbations prior to the diagnosis of COPD were identified on the basis of prescription of oral corticosteroids and/or a course of antibiotics within 3 days of a lower respiratory consultation, or a hospital attendance, as in previous studies [13]. Some of these may have been simple LRTIs, but as the patient was subsequently diagnosed with COPD and they required medication we are confident that they represent undiagnosed exacerbations. Some events may not have been recorded in patients' medical records if they did not consult regarding their symptoms, but similar issues arise in diagnosed patients.

### Conclusions

Our data show that having had just one moderate exacerbation in the year prior to diagnosis significantly increases the risk of future exacerbations but the risk is greater still in patients who had two or more moderate exacerbations. Placing patients with 0 or 1 moderate in the same low risk of future exacerbation groups (A and B) in the GOLD recommendations is not justified on the basis of our data and whether patients with a single moderate exacerbation should be included in the higher risk group E needs consideration. The impact of even a single exacerbation is now well recognised [29, 30] and we believe that the threshold for being in group E in the GOLD recommendations on initial pharmacotherapy should be amended to include patients with a single moderate exacerbation.

## Shareable PDF

10.1183/13993003.02240-2023.Shareable1 This one-page PDF can be shared freely online.
Shareable PDF ERJ-02240-2023.Shareable

## Supplementary material

**Please note:** supplementary material is not edited by the Editorial Office, and is uploaded as it has been supplied by the author. Supplementary material ERJ-02240-2023.Supplement
