# Supplementary material for: Exacerbation history and blood eosinophil count prior to diagnosis of COPD and risk of subsequent exacerbations
Source: Eur Respir J. 2024 Oct 3;64(4):2302240. doi: 10.1183/13993003.02240-2023 (PMC11447287; doi:10.1183/13993003.02240-2023)
Supplement: Supplementary file 1 [file ERJ-02240-2023.Shareable.pdf]

EUROPEAN RESPIRATORY JOURNAL
ORIGINAL RESEARCH ARTICLE
D.M.G. HALPIN ET AL.

SHAREABLE PDF

Exacerbation history and blood eosinophil count prior to diagnosis of COPD and risk of subsequent exacerbations

David M.G. Halpin, Heath Healey, Derek Skinner, Victoria Carter, Rachel Pullen and David Price

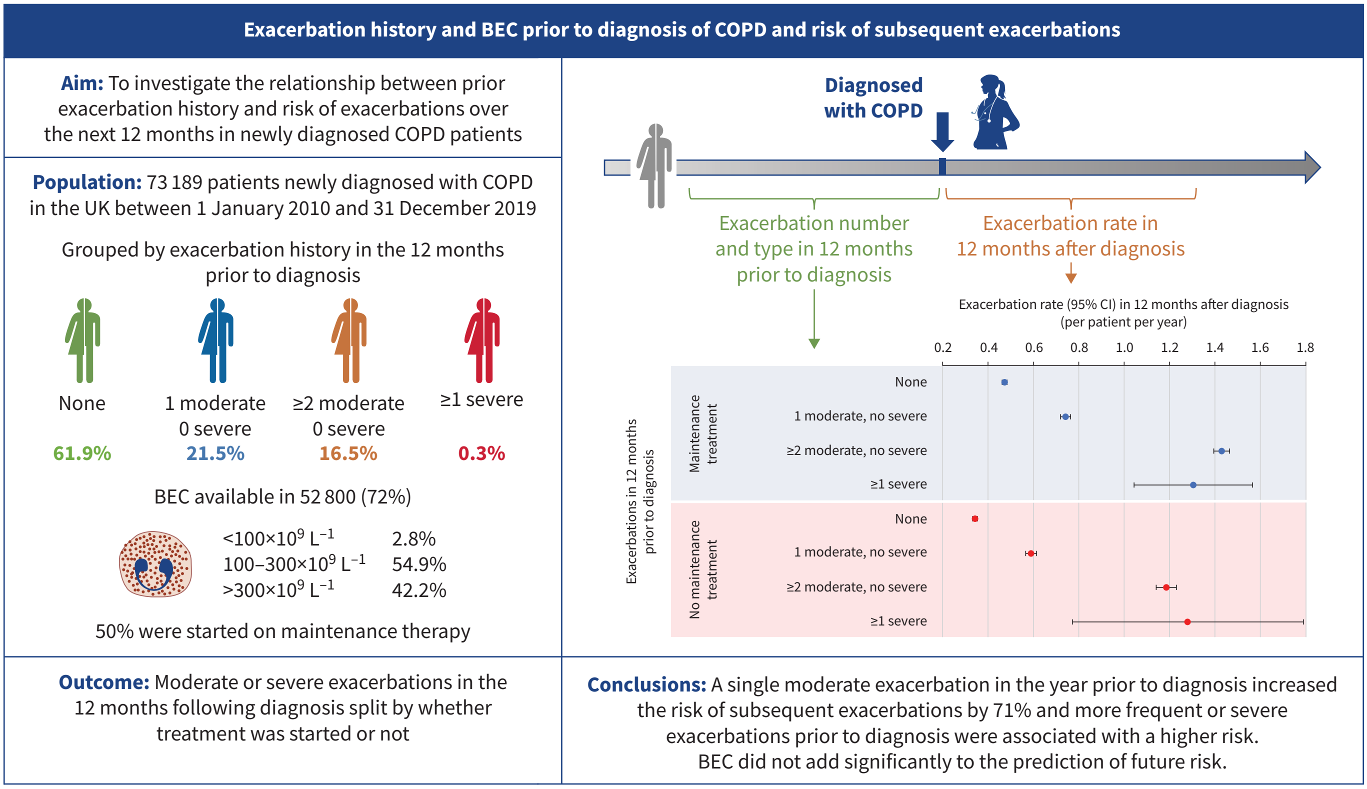

GRAPHICAL ABSTRACT Overview of the study. BEC: blood eosinophil count.

Link to published version: https://doi.org/10.1183/13993003.02240-2023

EUROPEAN RESPIRATORY JOURNAL
ORIGINAL RESEARCH ARTICLE
D.M.G. HALPIN ET AL.

SHAREABLE PDF

Exacerbation history and blood eosinophil count prior to diagnosis of COPD and risk of subsequent exacerbations

David M.G. Halpin [1,2], Heath Healey[3], Derek Skinner[3], Victoria Carter[3], Rachel Pullen[2] and David Price [2,3,4]

[1]University of Exeter Medical School, College of Medicine and Health, University of Exeter, Exeter, UK. [2]Observational and Pragmatic Research Institute, Singapore, Singapore. [3]Optimum Patient Care, Cambridge, UK. [4]Centre of Academic Primary Care, Division of Applied Health Sciences, University of Aberdeen, Aberdeen, UK.

Corresponding author: David M.G. Halpin (d.m.g.halpin@ex.ac.uk)

Check for updates

Shareable abstract (@ERSpublications)

In newly diagnosed patients, a single exacerbation prior to diagnosis of COPD is associated with a significant risk of exacerbations over the next 12 months, and more frequent or severe exacerbations prior to diagnosis are associated with a higher risk https://bit.ly/4fhTSOm

Cite this article as: Halpin DMG, Healey H, Skinner D, *et al*. Exacerbation history and blood eosinophil count prior to diagnosis of COPD and risk of subsequent exacerbations. *Eur Respir J* 2024; 64: 2302240 [DOI: 10.1183/13993003.02240-2023].

This extracted version can be shared freely online.

Copyright ©The authors 2024.

This version is distributed under the terms of the Creative Commons Attribution Licence 4.0.

This article has an editorial commentary: https://doi.org/10.1183/13993003.01019-2024

Received: 14 Dec 2023
Accepted: 19 July 2024

Abstract

Background Prior exacerbation history is used to guide initial maintenance therapy in COPD; however, the recommendations were derived from patients already diagnosed and treated.

Methods We assessed the rates of moderate (*i.e.* treated with antibiotics and/or systemic corticosteroids) and severe (*i.e.* hospitalised) exacerbations in the year following diagnosis in patients newly diagnosed with COPD according to their prior history of exacerbations, blood eosinophil count (BEC) and whether maintenance therapy was started. Data were extracted from the Optimum Patient Care Research Database.

Results 73 189 patients were included. 61.9% had no exacerbations prior to diagnosis, 21.5% had 1 moderate, 16.5% had ⩾2 moderate and 0.3% had ⩾1 severe. 50% were started on maintenance therapy. In patients not started on maintenance therapy the rates of moderate exacerbations in the year after diagnosis in patients with no, 1 moderate, ⩾2 moderate and ⩾1 severe prior exacerbations were 0.34 (95% CI 0.33–0.35), 0.59 (95% CI 0.56–0.61), 1.18 (95% CI 1.14–1.23) and 1.21 (95% CI 0.73–1.69), respectively. Similar results were seen in patients started on maintenance therapy. BEC did not add significantly to the prediction of future exacerbation risk.

Conclusions A single moderate exacerbation in the year prior to diagnosis increases the risk of subsequent exacerbations, and more frequent or severe exacerbations prior to diagnosis are associated with a higher risk.
