# Supplementary material for: Exacerbation history and blood eosinophil count prior to diagnosis of COPD and risk of subsequent exacerbations
Source: Eur Respir J. 2024 Oct 3;64(4):2302240. doi: 10.1183/13993003.02240-2023 (PMC11447287; doi:10.1183/13993003.02240-2023)
Supplement: Supplementary file 2 [file ERJ-02240-2023.Supplement.pdf]

On line supplement

Study variables and outcomes

For eligible patients the following data were extracted from the OPCRD: age at diagnosis; sex; body mass index (BMI, kg/m2) within 2 years of diagnosis; smoking status as recorded nearest to diagnosis (before or after) (categorised as current smokers, former smokers and never smokers); mMRC dyspnoea score; maximum eosinophil reading within 1 year of initial COPD diagnosis (continuous values and categorised as <150 x 10^9/L 150-300 x 10^9/L and >300 x 10^9/L); COPD Assessment Test (1)(CAT) score (categorised as normal 0-<6, low 6-<10, medium 10-<21, high 21-<31 and very high 31-40); GOLD group at diagnosis A, B or E (2), based on number of exacerbations and mMRC scores; number of moderate exacerbations in the 12 months prior to diagnosis (defined as events requiring a prescription of oral corticosteroids and/or a course of antibiotics within 3 days of a lower respiratory consultation, or a hospital attendance); number of severe exacerbations in the 12 months prior to diagnosis (defined as an admission to hospital for a respiratory related cause); maintenance treatment started at diagnosis (categorised as none, LABA, LAMA, LABA/ICS, LABA/LAMA and LABA/LAMA/ICS); number of moderate exacerbations in the 12 months following diagnosis; number of severe exacerbations in the 12 months following diagnosis; asthma, hypertension, ischaemic heart disease, heart failure, chronic kidney disease, type 2 diabetes, osteoporosis, depression or anxiety present at the time of, or prior to diagnosis.

Number of exacerbations in 12 months prior to diagnosis	Not started on maintenance therapy								Started on maintenance therapy							
	None		1 moderate, no severe		2 or more moderate, no severe		1 or more severe		None		1 moderate, no severe		2 or more moderate, no severe		1 or more severe	
Number of patients	20,938		6,240		3,878		43		24,267		9,477		8,185		161	
**Age**	mean	SD	mean	SD	mean	SD	mean	SD	mean	SD	mean	SD	mean	SD	mean	SD
	66.4	10.4	66.3	10.3	66.7	10.5	64.6	11.9	66.4	10.7	65.9	10.8	66.0	11.2	64.7	11.5
**Gender**	n	%	n	%	n	%	n	%	n	%	n	%	n	%	n	%
Male	11,939	57.3%	3,275	52.8%	1,818	47.1%	24	57.1%	13,591	56.3%	4,955	52.7%	3,857	47.4%	75	46.6%
Female	8,911	42.7%	2,929	47.2%	2,040	52.9%	18	42.9%	10,545	43.7%	4,451	47.3%	4,280	52.6%	86	53.4%
Other/Missing	88	0.4%	36	0.6%	20	0.5%	1	2.3%	131	0.5%	71	0.7%	48	0.6%		0.0%
**BMI**[1]	mean	SD	mean	SD	mean	SD	mean	SD	mean	SD	mean	SD	mean	SD	mean	SD
	26.7	5.6	27.0	5.7	27.4	6.0	28.7	5.3	27.4	6.0	27.4	6.2	28.0	6.2	28.2	7.7
missing (n (%)	1,155	5.5%	350	5.6%	184	4.7%	5	11.6%	1,118	4.6%	438	4.6%	340	4.2%	4	2.5%
**Smoking**[2]	n	%	n	%	n	%	n	%	n	%	n	%	n	%	n	%
Current	5,639	29.3%	1,673	29.7%	850	24.1%	9	24.3%	5,578	25.1%	2,108	24.3%	1,538	20.5%	30	20.8%
Ex	11,791	61.3%	3,466	61.6%	2,305	65.4%	24	64.9%	14,080	63.3%	5,611	64.7%	4,888	65.1%	89	61.8%
Non	1,817	9.4%	486	8.6%	371	10.5%	4	10.8%	2,586	11.6%	954	11.0%	1,086	14.5%	25	17.4%
Missing	1,691	8.1%	615	9.9%	352	9.1%	6	14.0%	2,023	8.3%	804	8.5%	673	8.2%	17	10.6%
**GOLD Stage**[3]	n	%	n	%	n	%	n	%	n	%	n	%	n	%	n	%
1 Mild	157	1.3%	58	1.5%	48	2.1%	0	0.0%	439	3.0%	184	3.1%	134	2.7%	3	3.4%
2 Moderate	1,474	12.1%	494	12.9%	325	14.0%	2	11.1%	2,942	20.2%	1,226	20.9%	1,007	20.1%	18	20.5%
3 Severe	6,920	57.0%	2,290	59.9%	1,382	59.6%	13	72.2%	8,288	56.9%	3,391	57.9%	2,919	58.2%	57	64.8%
4 Very Severe	3,586	29.5%	978	25.6%	565	24.4%	3	16.7%	2,898	19.9%	1,058	18.1%	955	19.0%	10	11.4%
missing	8,801	42.0%	2,420	38.8%	1,558	40.2%	25	58.1%	9,700	40.0%	3,618	38.2%	3,170	38.7%	73	45.3%
**GOLD Group**[4]	n	%	n	%	n	%	n	%	n	%	n	%	n	%	n	%
A	14,779	77.4%	4,432	77.6%					15,040	66.1%	5,931	66.4%				
B	4,321	22.6%	1,278	22.4%					7,726	33.9%	2,955	33.6%				
E					3,555	100%	40	100%					7,702	100%	150	100%
missing	1,838	8.8%	530	8.5%	323	8.3%	3	7.0%	1,501	6.2%	551	5.8%	483	5.9%	11	6.8%
**mMRC**[5]	n	%	n	%	n	%	n	%	n	%	n	%	n	%	n	%
1	6,051	31.7%	1,837	32.2%	976	27.5%	9	22.5%	4,452	19.6%	1,770	19.8%	1,346	17.5%	17	11.3%
2	8,519	44.6%	2,548	44.6%	1,615	45.5%	15	37.5%	10,258	45.1%	4,049	45.4%	3,438	44.7%	59	39.3%

3	3,327	17.4%	984	17.2%	679	19.1%	8	20.0%	5,633	24.8%	2,196	24.6%	2,029	26.4%	44	29.3%
4	1,044	5.5%	297	5.2%	235	6.6%	5	12.5%	2,100	9.2%	797	8.9%	762	9.9%	25	16.7%
5	139	0.7%	42	0.7%	48	1.4%	3	7.5%	310	1.4%	106	1.2%	118	1.5%	5	3.3%
missing	1,858	8.9%	532	8.5%	325	8.4%	3	7.0%	1,514	6.2%	559	5.9%	492	6.0%	11	6.8%
**CAT**[6]	n	%	n	%	n	%	n	%	n	%	n	%	n	%	n	%
Normal: 0 <6	808	23.8%	188	20.3%	116	19.3%	2	25.0%	701	3.0%	267	15.1%	156	10.5%	3	6.4%
Low: 6 <10	814	24.0%	251	27.0%	120	19.9%	2	25.0%	922	3.9%	341	19.3%	252	17.0%	8	17.0%
Medium: 10 <21	1,341	39.5%	363	39.1%	237	39.4%	3	37.5%	21,140	89.4%	789	44.7%	688	46.4%	24	51.1%
High: 21 <31	371	10.9%	109	11.7%	107	17.8%	1	12.5%	730	3.1%	303	17.1%	305	20.6%	7	14.9%
Very high: 31 <41	64	1.9%	17	1.8%	22	3.7%	0	0.0%	163	0.7%	67	3.8%	82	5.5%	5	10.6%
missing	17,540	83.8%	5312	85.1%	3,276	84.5%	35	81.4%	19,611	80.8%	7,710	81.4%	6,702	81.9%	114	70.8%
**Blood Eosinophil Count (x 10^9/L)**[7]	n	%	n	%	n	%	n	%	n	%	n	%	n	%	n	%
0 <100	459	3.1%	161	3.7%	75	2.7%	1	3.1%	506	2.9%	180	2.6%	144	2.4%	5	4.2%
100 - 300	8,584	57.3%	2,388	55.0%	1471	52.1%	16	50.0%	9,797	55.6%	3,615	53.0%	3,063	50.6%	69	58.5%
>300	5,936	39.6%	1,795	41.3%	1279	45.3%	15	46.9%	7,332	41.6%	3,023	44.3%	2,842	47.0%	44	37.3%
missing	5,959	28.5%	1,896	30.4%	1,053	27.2%	11	25.6%	6,632	27.3%	2,659	28.1%	2,136	26.1%	43	26.7%
**Initial Treatment**[8]									n	%	n	%	n	%	n	%
LABA									2,012	8.3%	774	8.2%	636	7.8%	14	8.7%
LAMA									10,309	42.5%	4,160	43.9%	3,572	43.6%	67	41.6%
LABA/ICS									7,654	31.5%	3,132	33.0%	3,417	41.7%	75	46.6%
LABA/LAMA									1,703	7.0%	557	5.9%	322	3.9%	6	3.7%
LABA/LAMA/ICS									118	0.5%	51	0.5%	50	0.6%	1	0.6%
None of the above[9]									4,027	16.6%	1,458	15.4%	1,026	12.5%	23	14.3%
**Prior Prevalence of Comorbidities**[10]	n	%	n	%	n	%	n	%	n	%	n	%	n	%	n	%
Asthma	2,632	12.6%	973	15.6%	1008	26.0%	26	60.5%	7,356	30.3%	3,313	35.0%	3,942	48.2%	124	77.0%
Hypertension	1,279	6.1%	457	7.3%	483	12.5%	16	37.2%	3,533	14.6%	1,575	16.6%	1,968	24.0%	58	36.0%
Ischaemic Heart Disease	277	1.3%	124	2.0%	131	3.4%	2	4.7%	710	2.9%	338	3.6%	428	5.2%	10	6.2%
Heart Failure	264	1.3%	100	1.6%	128	3.3%	3	7.0%	702	2.9%	356	3.8%	421	5.1%	14	8.7%
Chronic Kidney Disease	484	2.3%	171	2.7%	176	4.5%	1	2.3%	1,251	5.2%	549	5.8%	697	8.5%	15	9.3%
Type 2 Diabetes	467	2.2%	203	3.3%	213	5.5%	3	7.0%	1,312	5.4%	559	5.9%	829	10.1%	25	15.5%
Osteoporosis	276	1.3%	103	1.7%	159	4.1%	1	2.3%	773	3.2%	391	4.1%	579	7.1%	23	14.3%
Depression or Anxiety	1,248	6.0%	510	8.2%	567	14.6%	13	30.2%	3,430	14.1%	1,743	18.4%	2,208	27.0%	69	42.9%

Supplementary Table 1. Baseline characteristics by prior exacerbation history and whether maintenance therapy was started. [1] BMI Recorded or calculated BMI nearest (within 2 years) to initial COPD diagnosis; [2] Smoking nearest (before or after) initial COPD diagnosis; [3] GOLD 2022 Group based on FEV percent predicted (within 5 years prior); [4] GOLD 2023 Stages A, B, E; [5] mMRC Modified Medical Research Council) Dyspnoea Scale (within 1 year) [6] COPD Assessment Test (CAT) Score nearest (within 1 year) to initial COPD diagnosis; [7] Maximum eosinophil reading within 1 year of initial COPD diagnosis; [8] Patients may receive multiple drugs on the same "initial treatment" day. Combination product counts don't include multiple single items on the same day, [9] Patients classed as none may receive other drug classes not included in this list e.g. SABA. [10] Prior Prevalence – prevalence of conditions diagnosed before initial COPD diagnosis.

		Maintenance Treatment including ICS			Maintenance Treatment not including ICS		
Exacerbation History in year prior to diagnosis		Number of patients	moderate or severe exacerbations in year following diagnosis		Number of patients	moderate or severe exacerbations in year following diagnosis	
None	All	12,301	Rate (95% CI)	0.51 (0.49 - 0.53)	11,966	Rate (95% CI)	0.44 (0.42 - 0.46)
1 moderate, no severe	All	4,871	Rate (95% CI)	0.78 (0.74 - 0.81)	4,606	Rate (95% CI)	0.70 (0.66 - 0.75)
			IRR v none (95% CI)	1.53 (1.43 - 1.63)		IRR v none (95% CI)	1.62 (1.51 - 1.74)
	Eos <100	73	Rate (95% CI)	0.60 (0.38 - 0.83)	107	Rate (95% CI)	0.75 (0.44 - 1.06)
			IRR v none (95% CI)	1.20 (0.72 – 2.00)		IRR v none (95% CI)	3.18 (1.81 - 5.59)
	Eos 100-300	1,789	Rate (95% CI)	0.81 (0.75 - 0.88)	1,826	Rate (95% CI)	0.64 (0.58 - 0.70)
			IRR v none (95% CI)	1.61 (1.46 - 1.79)		IRR v none (95% CI)	1.53 (1.37 - 1.72)
	Eos > 300	1,595	Rate (95% CI)	0.84 (0.77 - 0.91)	1,424	Rate (95% CI)	0.80 (0.72 - 0.87)
			IRR v none (95% CI)	1.49 (1.33 - 1.66)		IRR v none (95% CI)	1.64 (1.45 - 1.85)
2 or more moderate, no severe	All	4,743	Rate (95% CI)	1.52 (1.46 - 1.59)	3,442	Rate (95% CI)	1.30 (1.23 - 1.36)
			IRR v none (95% CI)	3.00 (2.83 - 3.18)		IRR v none (95% CI)	2.97 (2.77 - 3.19)
	Eos <100	91	Rate (95% CI)	1.78 (1.31 - 2.25)	53	Rate (95% CI)	1.06 (0.56 - 1.55)
			IRR v none (95% CI)	3.54 (2.43 - 5.17)		IRR v none (95% CI)	4.49 (2.29 - 8.82)
	Eos 100-300	1,759	Rate (95% CI)	1.50 (1.40 - 1.61)	1,304	Rate (95% CI)	1.18 (1.08 - 1.28)
			IRR v none (95% CI)	2.95 (2.72 - 3.28)		IRR v none (95% CI)	2.80 (2.50 - 3.14)
	Eos > 300	1,636	Rate (95% CI)	1.64 (1.53 - 1.75)	1,206	Rate (95% CI)	1.50 (1.38 - 1.63)
			IRR v none (95% CI)	2.92 (2.64 - 3.22)		IRR v none (95% CI)	3.08 (2.74 - 3.47)
1 or more severe	All	106	Rate (95% CI)	1.35 (0.94 - 1.76)	55	Rate (95% CI)	1.22 (0.57 - 1.87)
			IRR v none (95% CI)	2.65 (1.97 - 3.58)		IRR v none (95% CI)	2.79 (1.78 - 4.40)
	Eos <100	2	Rate (95% CI)	0.50 (0.00 - 1.81)	3	Rate (95% CI)	0.67 (0.00 - 1.56)
			IRR v none (95% CI)	1.00 (0.06 - 17.0)		IRR v none (95% CI)	2.84 (0.20 - 39.8)
	Eos 100-300	40	Rate (95% CI)	1.18 (0.64 - 1.71)	29	Rate (95% CI)	1.14 (0.27 – 2.00)
			IRR v none (95% CI)	2.33 (1.41 - 3.85)		IRR v none (95% CI)	2.71 (1.44 - 5.09)
	Eos > 300	32	Rate (95% CI)	1.68 (1.03 - 2.34)	12	Rate (95% CI)	1.75 (0.00 - 3.75)
			IRR v none (95% CI)	2.95 (2.06 - 4.58)		IRR v none (95% CI)	3.59 (1.47 - 8.79)

Supplementary Table 2. Rate of moderate and severe exacerbations in the 12 months following diagnosis and incidence rate ratio (IRR) compared to patients with no prior exacerbations, in patients started on maintenance therapy including ICS or not including ICS, according to history of exacerbations in the 12 months prior to diagnosis and blood eosinophil (Eos) counts (x 10^9/L). CI – Confidence Interval.

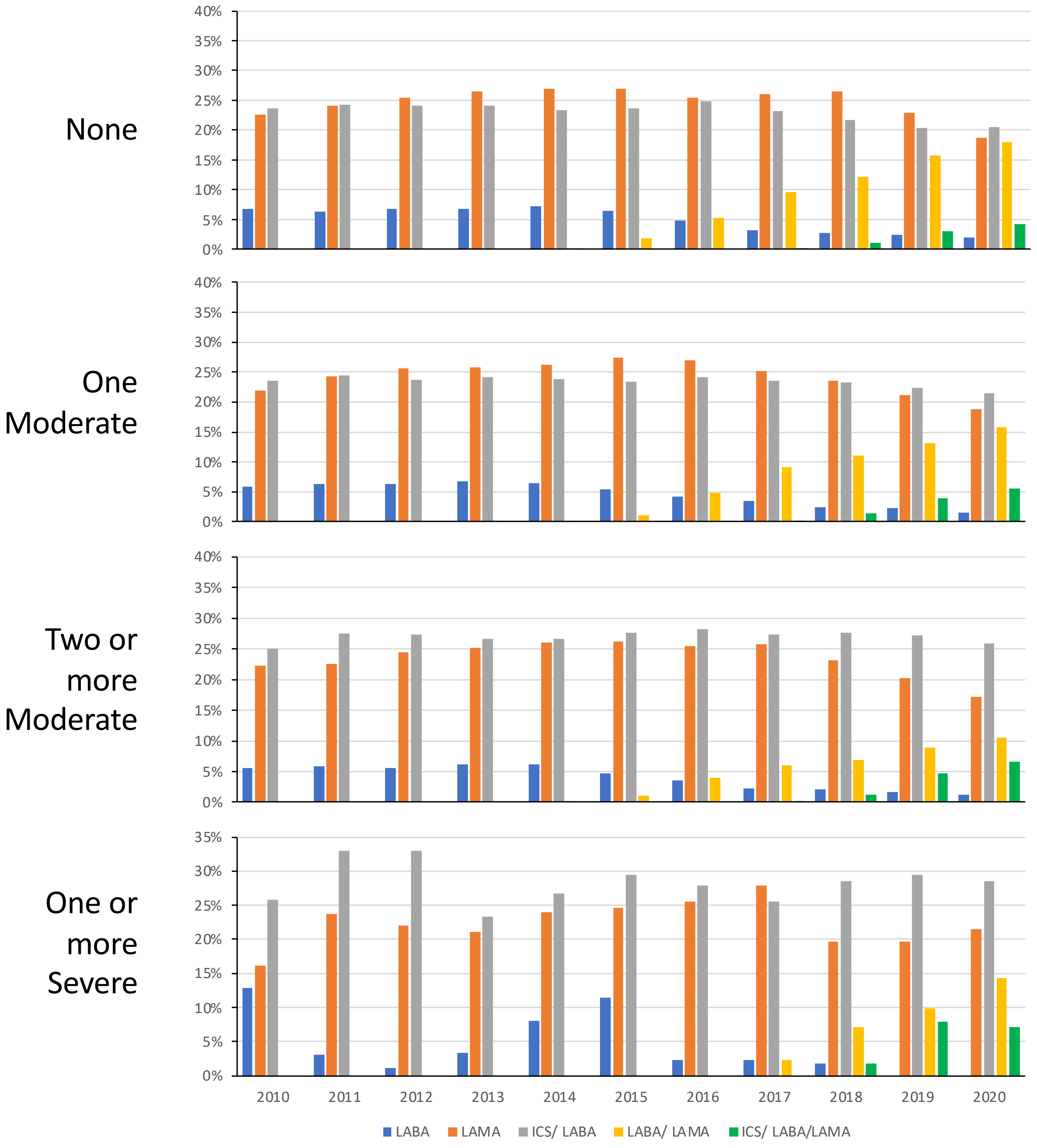

Supplementary Figure 1. Proportion of patients started on maintenance therapies by year according to prior exacerbation history. Patients may have received multiple drugs on the same "initial treatment" day. Combination product counts don’t include multiple single items.

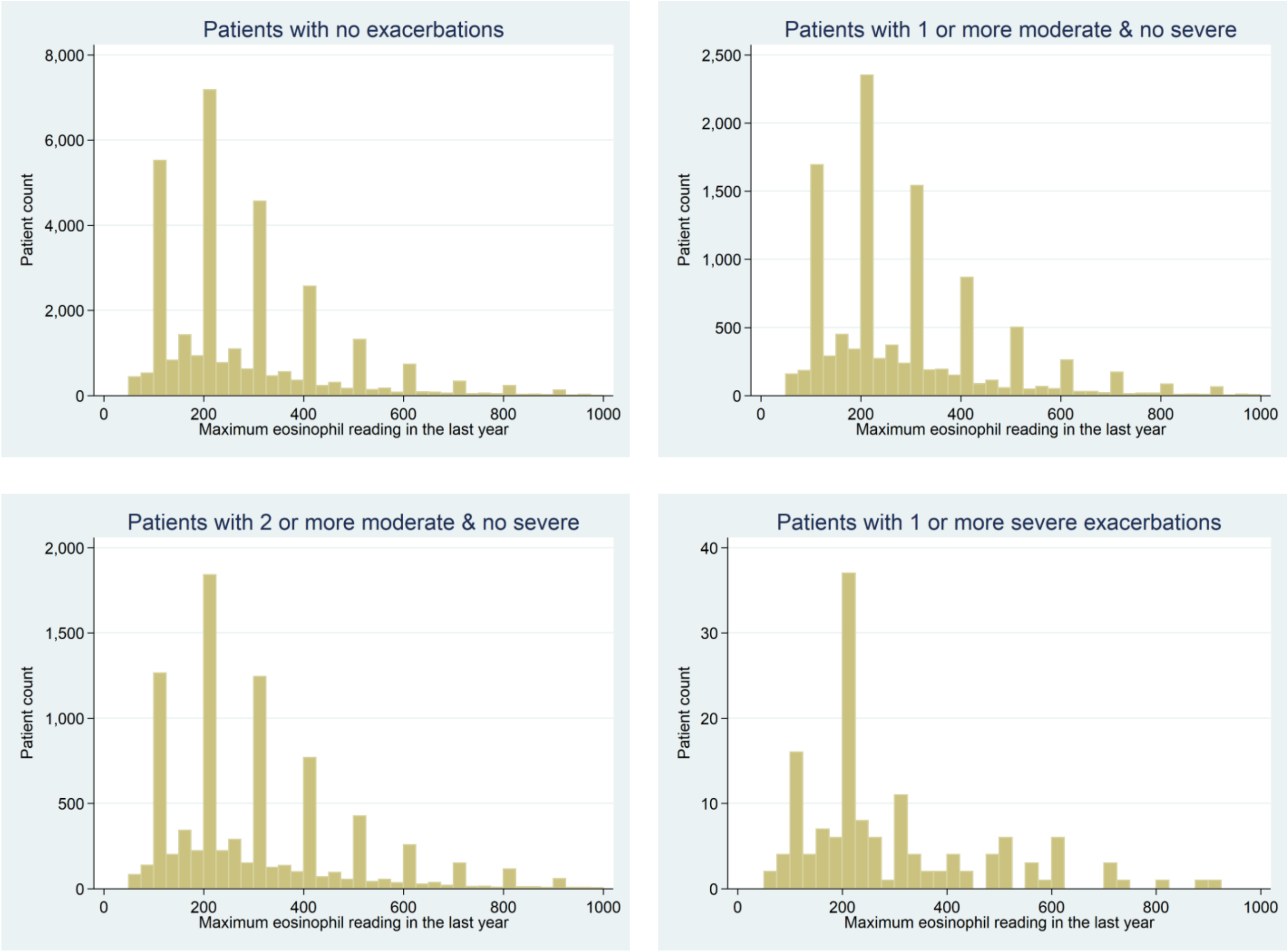

Supplementary Figure 2. Distribution of blood eosinophil counts in each of the prior exacerbation groups.

References

1. Jones PW, Harding G, Berry P, et al. Development and first validation of the COPD Assessment Test. Eur Respir J 2009: 34: 648-654

2. Agustí A, Celli BR, Criner GJ, Halpin D, Anzueto A, Barnes P, et al. Global Initiative for Chronic Obstructive Lung Disease 2023 Report: GOLD Executive Summary. Eur Respir J. 2023;61(4).
